# Congenital Hepatic Fibrosis in the Franches-Montagnes Horse Is Associated with the Polycystic Kidney and Hepatic Disease 1 (*PKHD1*) Gene

**DOI:** 10.1371/journal.pone.0110125

**Published:** 2014-10-08

**Authors:** Michaela Drögemüller, Vidhya Jagannathan, Monika M. Welle, Claudia Graubner, Reto Straub, Vinzenz Gerber, Dominik Burger, Heidi Signer-Hasler, Pierre-André Poncet, Stéphane Klopfenstein, Ruedi von Niederhäusern, Jens Tetens, Georg Thaller, Stefan Rieder, Cord Drögemüller, Tosso Leeb

**Affiliations:** 1 Institute of Genetics, University of Bern, Bern, Switzerland; 2 Swiss Competence Center of Animal Breeding and Genetics, University of Bern, Bern University of Applied Sciences HAFL and Agroscope, Bern, Switzerland; 3 Institute of Animal Pathology, University of Bern, Bern, Switzerland; 4 Swiss Institute of Equine Medicine, University of Bern and Agroscope, Bern, Switzerland; 5 Bern University of Applied Sciences HAFL, Zollikofen, Switzerland; 6 HIPPOP, Lignerolle, Switzerland; 7 Swiss Franches-Montagnes Breeding Association, Avenches, Switzerland; 8 Agroscope, Swiss National Stud Farm, Avenches, Switzerland; 9 Institute of Animal Breeding and Husbandry, Christian-Albrechts-University, Kiel, Germany; CSIRO, Australia

## Abstract

Congenital hepatic fibrosis has been described as a lethal disease with monogenic autosomal recessive inheritance in the Swiss Franches-Montagnes horse breed. We performed a genome-wide association study with 5 cases and 12 controls and detected an association on chromosome 20. Subsequent homozygosity mapping defined a critical interval of 952 kb harboring 10 annotated genes and loci including the polycystic kidney and hepatic disease 1 (autosomal recessive) gene (*PKHD1*). *PKHD1* represents an excellent functional candidate as variants in this gene were identified in human patients with autosomal recessive polycystic kidney and hepatic disease (ARPKD) as well as several mouse and rat mutants. Whereas most pathogenic *PKHD1* variants lead to polycystic defects in kidney and liver, a small subset of the human ARPKD patients have only liver symptoms, similar to our horses with congenital hepatic fibrosis. The *PKHD1* gene is one of the largest genes in the genome with multiple alternative transcripts that have not yet been fully characterized.

We sequenced the genomes of an affected foal and 46 control horses to establish a comprehensive list of variants in the critical interval. We identified two missense variants in the *PKHD1* gene which were strongly, but not perfectly associated with congenital hepatic fibrosis. We speculate that reduced penetrance and/or potential epistatic interactions with hypothetical modifier genes may explain the imperfect association of the detected *PKHD1* variants. Our data thus indicate that horses with congenital hepatic fibrosis represent an interesting large animal model for the liver-restricted subtype of human ARPKD.

## Introduction

Autosomal recessive polycystic kidney disease (ARPKD) has an incidence of about 1:20,000 in humans [1]. It is caused by variants in the polycystic kidney and hepatic disease 1 (autosomal recessive) gene (*PKHD1*). More than 500 different potentially pathogenic variants in the human *PKHD1* gene have been described, and there is considerable variation in the clinical phenotype and age of onset ([1], [2], http://www.humgen.rwth-aachen.de/index.php). Patients carrying two truncating *PKHD1* mutations typically die perinatally, whereas patients with residual *PKHD1* function typically have less severe disease, which predominantly affects the kidney and the liver. A small subset of patients with *PKHD1* mutations only show hepatic disease [2], [3].

The PKHD1 protein probably has an essential role in the primary cilia of tubular epithelial cells, which are required as mechanosensors and regulate Ca^2+^ influx. PKHD1 probably directly interacts with polycystin 2 (PKD2), but its precise function and the role of its many different isoforms are unknown [4]. The *PKHD1* gene is very complex and spans about 500 kb with multiple alternative transcripts. The best studied human *PKHD1* transcript comprises 67 exons, has an open reading frame of 12,222 nucleotides, and encodes a protein of 4,074 amino acids, which has also been termed fibrocystin or polyductin [5], [6].

Several rodent models of ARPKD have been described. The polycystic rat (PCK) carries a spontaneous splice site mutation in the *Pkhd1* gene and is characterized by cyst formation in kidney and liver. There are also several targeted mouse mutants available including one that exclusively shows cystic biliary dysgenesis without kidney alterations [7]–[10]. The occurrence of similar diseases affecting exclusively the liver has been reported in monkey [11], and different sporadic cases in domestic animal species like cattle [12], [13], cat [14], dog [15], and horse [16], [17]. We previously identified 30 foals with liver lesions compatible with congenital hepatic fibrosis in a retrospective study [17], [18]. Anamnestic data revealed clinical signs of severe liver injury in most affected animals [16], [17]. Pathologic examination showed severely enlarged, firm livers with thin-walled cysts. Histologically, the livers showed diffuse porto-portal bridging fibrosis with many small, irregularly formed and sometimes cystic bile ducts [18]. All foals belonged to the Swiss Franches-Montagnes breed. Pedigree analysis revealed that the affected animals could be traced back to one stallion [18]. These results strongly suggest that congenital hepatic fibrosis in Swiss Franches-Montagnes horses is an autosomal recessively inherited genetic defect.

During the last six years seven inbred foals were born with congenital liver fibrosis and submitted for genetic investigations. The aim of the present study was to identify the assumed genetic cause for the condition using a positional approach and next generation sequencing in order to develop a genetic test to prevent further at risk matings.

## Results

### Congenital liver fibrosis in Franches-Montagnes horses

**Figure 1:**
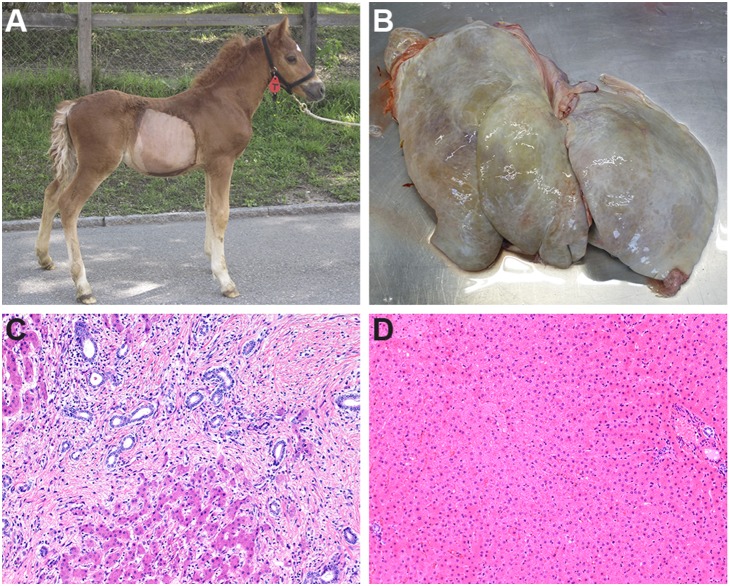
Congenital liver fibrosis in Franches-Montagnes horses. (A) Affected, 2 month old foal. The foal was small for its age and displayed a potbelly. (B) Enlarged and grey colored liver of the affected foal with macroscopically visible cysts. (C) Histological image of liver tissue from a foal with congenital hepatic fibrosis. Note the marked porto-portal bridging fibrosis and the abundant dilated bile ductules surrounded by some inflammatory cells within the fibrotic tissue (H&E, 400x). (D) Histological image of liver tissue from a non-affected foal. Note the small bile ducts in the portal areas and the absence of fibrosis (H&E 400x).

**Figure 2:**
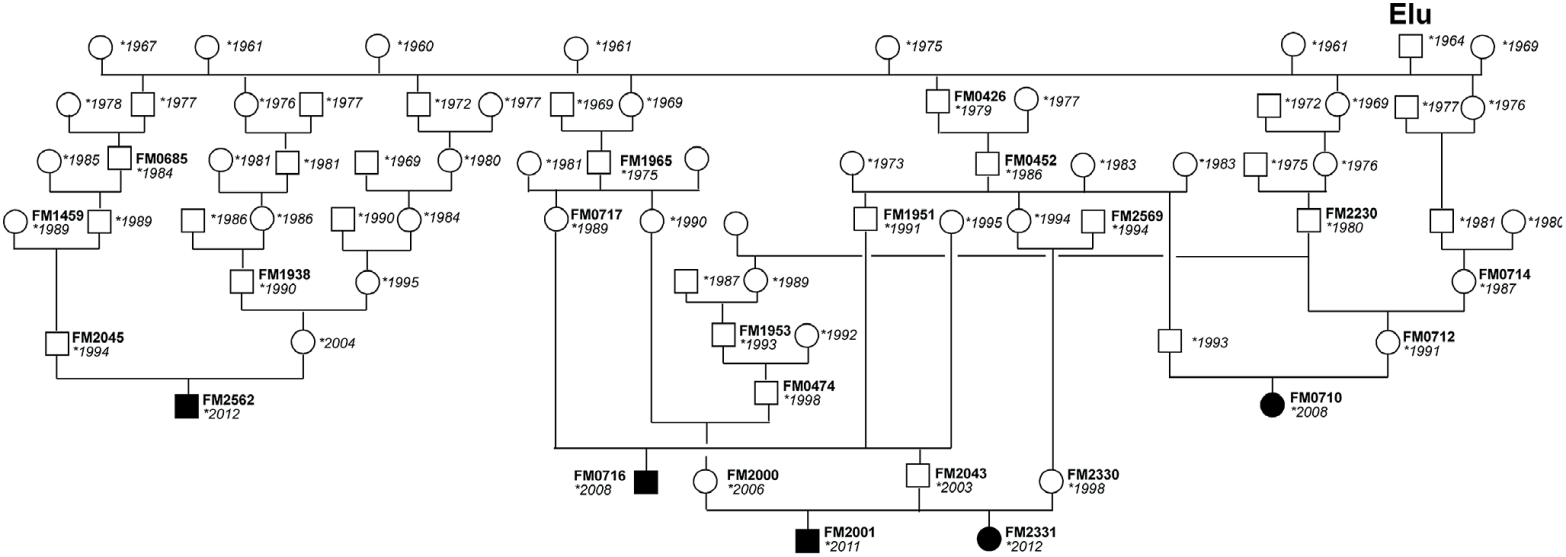
Pedigree of five Franches-Montagnes horses with congenital hepatic fibrosis. Males are represented by squares, females by circles. Affected animals are shown with black filled symbols, the year of birth is shown for all animals, and FM laboratory numbers are given for animals from which DNA was available. The causative genetic defect was probably mainly spread into the population by the stallion Elu born in 1964. All affected foals are inbred to this stallion and have him both as paternal and maternal ancestor.

Between the years 2008 and 2013, we recruited a total of 7 Franches-Montagnes foals, 5 males and 2 females, aged 3 weeks to 12 months, with clinical signs of severe liver injury (Figure 1). The parents of all cases were clinically inconspicuous. In 5 out of these 7 foals the quality of tissue or blood samples and the extracted DNA was sufficient for genotyping with SNP chips. The parents of these 5 foals could be traced, both on the maternal and the paternal lines over 3 to 7 generations, to a single common male ancestor (Elu) born in 1964 (Figure 2). Therefore, we concluded that a recessively inherited mutation was most likely, in agreement with our earlier studies [18]. Formalin-fixed paraffin-embedded liver samples from the earlier study were available and we successfully extracted DNA from 13 of these affected foals.

Two of the recent cases were admitted to the horse clinic of the University of Bern for in-depth clinical examination (Figure 1). They were in a reduced body shape and showed depressed behavior. Their abdomens were enlarged and feces were yellowish and of an oily consistence. Trans-abdominal ultrasound revealed an increased liver size with distinct cystic hypo-echoic lesions. Hematology showed a severe leukocytosis due to a severe neutrophilia. The laboratory chemistry showed extremely high liver bile acids. Both patients were euthanized and subjected to a necropsy.

Histopathological examination of all affected foals revealed that the liver showed diffuse porto-portal bridging fibrosis with many small, irregularly formed and sometimes cystic bile ducts (Figure 1C) similar to the earlier series of 30 reported cases of congenital liver fibrosis in the Franches-Montagnes breed [18].

### Mapping to a region on ECA 20 containing *PKHD1* as functional candidate gene

**Figure 3:**
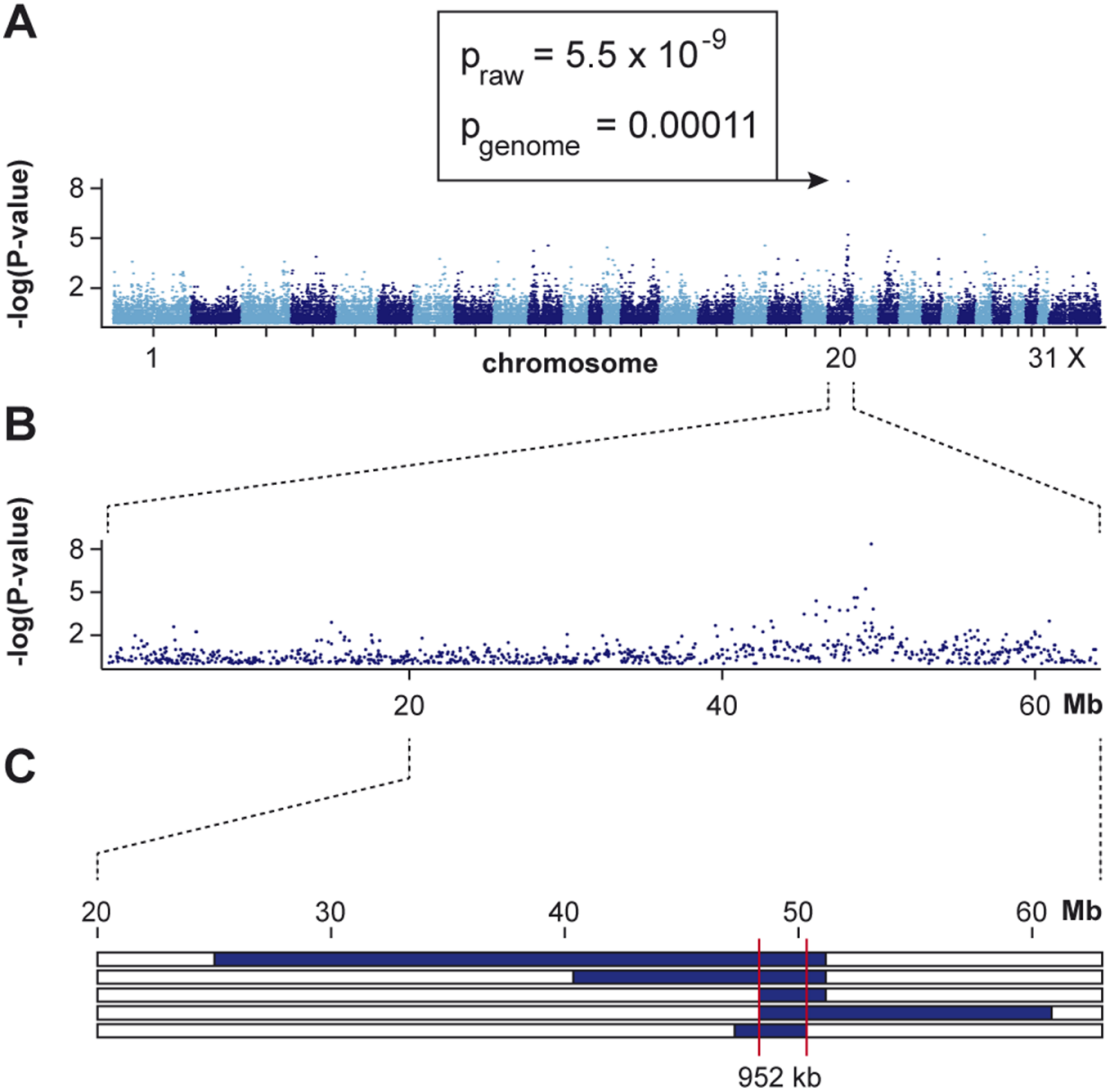
Mapping of congenital hepatic fibrosis to chromosome 20 with 5 cases and 12 controls. (**A**) Manhattan plot of the genome-wide allelic association study (GWAS). (**B**) Detailed view of the associated region. A single SNP at position 49,643,575 exceeded the genome-wide significance threshold. (**C**) Homozygosity mapping. The blue bars represent homozygous regions with shared alleles in the 5 affected foals. The common identical by descent segment among the 5 affected animals delineates a critical interval ranging from Chr20∶49,164,218–50,115,936.

We genotyped 65,157 SNPs on 5 affected foals and 12 non-affected Franches-Montagnes horses. After removing non-informative markers (MAF<0.1) and markers with call rates below 90%, we retained 38,394 SNPs for an allelic genome-wide association study (GWAS). The genomic inflation factor in this analysis was 1.33, indicating stratification due to the usage of closely related animals (Figure 2). The analysis revealed a single associated SNP, BIEC2-564517, at position 49,643,575 on chromosome 20. The raw p-value of the association was 5.5×10^−9^. This exceeds the Bonferroni-corrected significance threshold (p_Bonf_ = 0.00021) and also an empirical significance threshold determined after 100,000 permutations of the phenotypes (p_genome_ = 0.00011, Figure 3).

Based on the pedigree records we hypothesized that the affected foals were most likely inbred and related to a single founder animal (Figure 2). Under this scenario the affected individuals were expected to be identical by descent for the causative mutation and flanking chromosomal segments. We therefore analyzed the cases for the presence of a region of homozygosity with simultaneous allele sharing near the associated SNP on chromosome 20. In this region all 5 cases indeed were homozygous, and shared identical alleles over 31 consecutive SNP markers. This segment delineates a critical interval of 952 kb between the closest heterozygous markers on either side of the shared homozygous block (Chr20∶49,164,218–50,115,936; Figure 3C).

According to the NCBI annotation release 101 of the EquCab2 assembly the critical interval contains 6 annotated protein coding genes (*PKHD1*, *IL17A*, *IL17F*, *MCM3, PAQR8, EFHC1*), 2 microRNA genes (*MIR206-2*, *MIR133B*) and 2 uncharacterized loci (LOC102148758, LOC102148781). A careful inspection of these genes and database searches of their presumed function revealed the polycystic kidney and hepatic disease 1 gene (*PKHD1*) as an excellent functional candidate gene for congenital hepatic fibrosis in horses. Mutations in this gene cause autosomal recessive polycystic kidney (ARPKD) disease in humans, also known as polycystic kidney and hepatic disease 1 (OMIM#263200).

### Whole genome re-sequencing reveals no perfectly associated DNA variant

We opted to sequence the whole genome of one affected foal and an obligate heterozygous carrier sire of another case to generate a comprehensive list of variants with respect to the equine reference genome from a non-affected Thoroughbred horse.

Due to the recessive inheritance and the fatal effect of the mutation, we initially hypothesized that most likely a loss of function mutation affecting the coding sequence of *PKHD1* would be responsible for the congenital hepatic fibrosis phenotype. Within the mapped 952 kb interval, we identified 16 non-synonymous variants (Table 1; Figure S1). Two of these are most likely due to frame-shift errors in the genome reference sequence as the computer-predicted RefSeq transcript (XM_001498967.4) contains two additional nucleotides compared to the genome reference. The remaining 14 non-synonymous variants were all missense variants within the *PKHD1* gene. All 14 non-synonymous variants were homozygous in the sequenced case and heterozygous in the sequenced obligate carrier.

**Table 1:** Non-synonymous variants in the critical interval with respect to the EquCab 2 reference assembly of an affected Franches-Montagnes horse.

Position on chr.20	Referenceallele	Variant allele	Gene	Variant (cDNA)	Variant (protein)
49,398,500	C	T	*PKHD1*	c.10796G>A	p.R3599H
49,398,692	A	C	*PKHD1*	c.10604T>G	p.L3535R
49,597,760	A	T	*PKHD1*	c.6845T>A	p.I2282N
49,630,834	G	A	*PKHD1*	c.6112C>T	p.H2038Y
49,630,951	T	C	*PKHD1*	c.5995A>G	p.K1999E
49,709,730	C	A	*PKHD1*	c.4462T>G	p.A1488S
49,709,928	C	T	*PKHD1*	c.4264G>A	p.D1422N
49,710,356	G	C	*PKHD1*	c.3836C>G	p.A1279G
49,710,359	C	T	*PKHD1*	c.3833G>A	p.R1278Q
49,710,363	A	G	*PKHD1*	c.3829T>C	p.S1277P
49,710,468	C	T	*PKHD1*	c.3724G>A	p.E1242K
49,740,355	C	T	*PKHD1*	c.1811G>A	p.R604Q
49,741,254	C	T	*PKHD1*	c.1670G>A	p.R557Q
49,766,468	T	C	*PKHD1*	c.317A>G	p.E106G
50,077,556	G	GC	*EFHC1*	Probable error in the reference assembly	
50,077,559	G	GC	*EFHC1*	Probable error in the reference assembly	

We then compared the sequence data from the affected foal to whole genome sequences from 28 non-affected Franches-Montagnes horses and 18 horses of various other breeds (Table S1). All of the 14 non-synonymous variants also occurred in control horses of other breeds, 12 of them even in homozygous state. Two out of the 14 non-synonymous variants were only found in heterozygous state in the control horses.

We then focused on these two variants, *PKHD1:c.6112C>T* and *PKHD1:c.6845T>A*, predicted to cause the non-conservative changes p.H2038Y and p.I2282N on the protein level (Figure S2). We validated the variants by Sanger sequencing. We subsequently confirmed that all five affected foals were homozygous, while their parents were heterozygous compared to the reference sequence. We genotyped larger cohorts for these variants and noticed perfect linkage disequilibrium between the two variants in more than 300 investigated horses. We were unable to reliably genotype the *c.6112C>T* variant from our paraffin-embedded formalin-fixed and other low quality DNA samples. Therefore, we then genotyped only *c.6845T>A* in a very large cohort of more than 2,300 horses. We observed a strong, but not perfect association between *c.6845T>A* variant and the congenital hepatic fibrosis phenotype. We had 1 histopathologically confirmed case that carried the variant only in heterozygous state and we had 3 Franches-Montagnes horses that were homozygous for the variant, but did not show any clinical symptoms up to at least an age of 6 years (Table 2). Based on the perfect linkage disequilibrium observed between *c.6112C>T* and *c.6845T>A*, we speculate that the *c.6112C>T* variant would show the same or a highly similar association result (Table S2). We also encountered a New Forest Pony that was homozygous for both variants and not reported to show any signs of liver disease.

**Table 2:** Genotype distribution of 2 strongly associated *PKHD1* variants.

		g.49,597,760A>T			g.49,552,834C>T		
		c.6845T>A			intronic		
	Total	TT	AT	AA	CC	CT	TT
Affected Franches-Montagnes foals	20	-	1	19	-	3	17
Franches-Montagnes controls (≥5 years)	173	135	35	3	142	30	1
Franches-Montagnes population controls	1564	1304	259	1^a^	1346	217	1^a^
Total	1757^b^	1439	295	23	1488	250	19

a This horse was slaughtered before one year of age and the phenotype must be considered as unknown.

b This table summarizes a subset of all horses that were analyzed. In this table we list only those horses for which the genotypes at both variants were available.

As we did not find any non-synonymous variant with perfect association we then also investigated all other variants in the critical interval. The sequenced affected foal carried a total of 2,048 homozygous sequence variants compared to the reference genome (including the 16 non-synonymous variants of Table 1). The sequenced obligate carrier was heterozygous for 471 of these. Excluding variants that were homozygous in any of the 46 control genomes further reduced this list down to 64 variants (Table S2). Only one of these 64 variants was exclusively present in Franches-Montagnes horses. This variant, a single base transition, was located in the middle of an intron of *PKHD1* more than 4 kb away from the next exon boundary. We genotyped this variant in a large cohort of horses and found 3 cases, which were not homozygous for this variant compared to only 1 for the two non-synonymous *PKHD1* variants (Table 2).

As the horse genome reference sequence contains three gaps in the *PKHD1* gene we amplified these regions by PCR and analyzed the obtained products by Sanger sequencing. The comparison of data from affected and non-affected horses revealed no indication for additional variants located in these regions.

### RNA-seq and RT-PCR transcript analyses in liver

We then tested whether a non-coding variant or a variant in an unknown exon of the *PKHD1* gene might be responsible for the disease. Therefore, we performed an RNA-seq experiment using liver RNA from an affected and a control horse. We mapped the reads to the reference genome and produced a gene annotation of the equine *PKHD1* gene (Table S3). For numbering the exons in the horse genome we used the human *PKHD1* transcript isoform 1 as reference, which is derived from 67 exons in the human genome. All but one of the 67 human exons were also present in the horse. The human exon 42 was absent in the equine liver RNA-seq data and could also not be amplified by RT-PCR.

We additionally identified spliced reads indicating the presence of 9 previously unknown exons in the horse (Table S3). No disease associated sequence variant was located within these newly identified *PKHD1* exons. All 75 horse specific *PKHD1* exons were transcribed in both, the affected and the control animal. We did not assign the newly identified exons to different *PKHD1* transcripts as the *PKHD1* gene obviously encodes a variety of alternative spliced variants [5]. We also amplified approximately 90% of the *PKHD1* coding sequence as overlapping RT-PCR products from liver RNA of the case and the control. Subsequent Sanger sequencing of these RT-PCR products confirmed the results obtained by RNA-seq and did not indicate any structural defect in the *PKHD1* transcript from the affected horse.

### Allele specific transcript quantification in an obligate carrier

**Figure 4:**
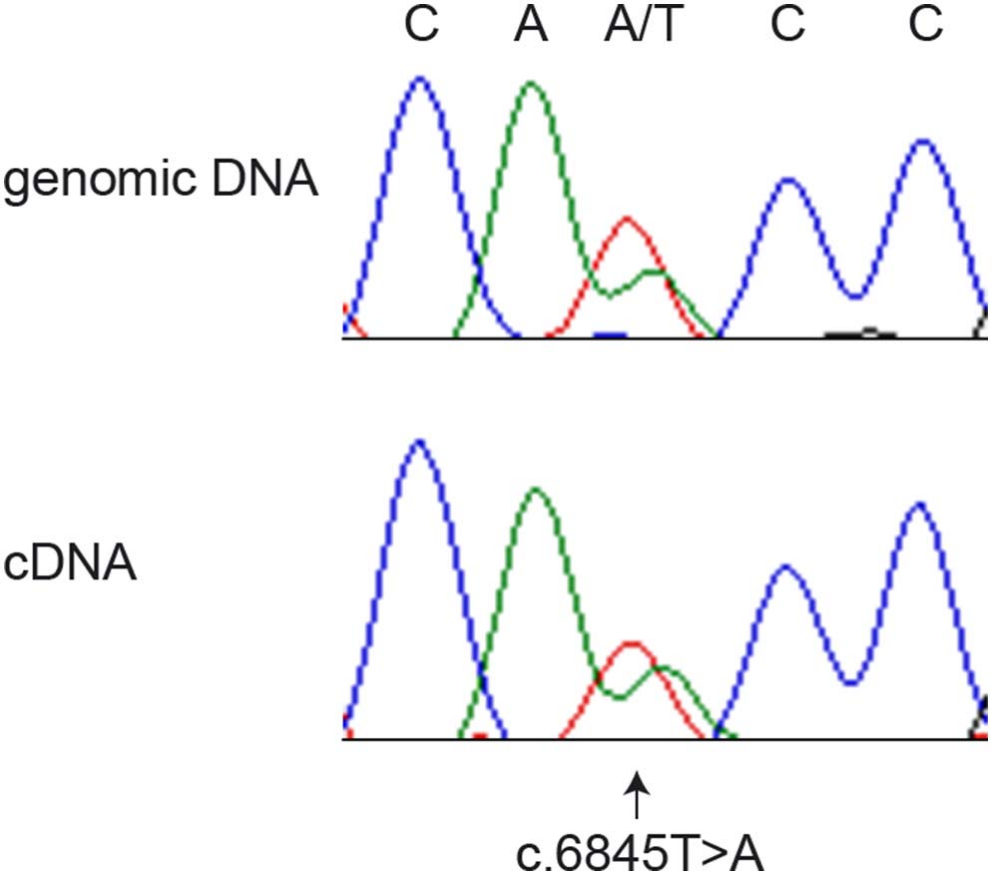
Allele-specific quantification of *PKHD1* transcripts. We sequenced a PCR product derived from genomic DNA and an RT-PCR product derived from liver RNA of a heterozygous stallion, which had already sired an affected foal and was therefore considered an obligate carrier of the disease causing variant. The electropherograms show comparable ratios of both alleles on genomic DNA and cDNA. This indicates that both alleles are transcribed at similar levels and that there is no nonsense-mediated decay of the transcript derived from the disease-associated A-allele.

As we could not identify a perfectly associated non-synonymous variant we investigated whether *PKHD1* transcripts of the disease-associated allele and the wildtype allele were present in equal amounts in an obligate carrier. We obtained a liver biopsy from an obligate carrier and compared the ratios between the two alleles from genomic DNA and from cDNA obtained by RT-PCR from liver RNA. This experiment confirmed the presence of approximately equal amounts of transcripts from both alleles (Figure 4).

## Discussion

Despite the successful mapping of the locus for congenital hepatic fibrosis in Franches-Montagnes horses to a genomic region containing a plausible functional candidate gene we could not identify a perfectly associated sequence variant.

If the phenotype in these horses is indeed caused by a defect in the *PKHD1* gene, it could either be a non-synonymous variant directly affecting the protein function or a non-coding regulatory variant affecting gene expression. We have systematically evaluated all non-synonymous variants and found two strongly associated variants, but for both of these variants we found rare non-affected horses that were also homozygous for these variants. Due to the strong association the *c.6845T>A* variant is currently used in marker-assisted selection for breeding decisions. Approximately 14% of the current Franches-Montagnes population are heterozygous for this variant and matings between heterozygous animals are currently forbidden. It has to be cautioned that not all exons of the *PKHD1* gene are known, but we evaluated at least all transcribed regions that we could identify after an RNA-seq experiment from liver RNA.

We also tried to systematically investigate all non-coding variants in the critical interval, which is of course challenging due to the size of the investigated region and an imperfect equine reference genome. Our data argue quite strongly against a regulatory causative variant for two reasons: On the one hand we did not find any variants that were stronger associated than the two non-synonymous variants. We only found one intronic variant with strong association, which was also private to the Franches-Montagnes breed. However, this variant was heterozygous in 2 of our cases, which were homozygous for both of the non-synonymous variants. This suggests that this intronic variant originated very recently by a mutation event on the disease-associated haplotype after it acquired the disease-causing mutation. On the other hand the quantitative analysis of allele-specific *PKHD1* transcripts from a heterozygous carrier animal did not show large differences in the amount of *PKHD1* mRNA from either the wildtype or the disease-associated allele, which also strongly argues against a regulatory variant affecting transcription.

These findings emphasize that congenital hepatic fibrosis in the Franches-Montagnes horses is probably not inherited in a strictly monogenic autosomal recessive mode with full penetrance. Possibly one or both of the associated missense sequence variants are pathogenic but not fully penetrant, e.g. due to unknown epistatic interactions in the genome.

Among more than 2,300 tested horses we only found 5 horses, which carried the potentially pathogenic variants in homozygous state and were not clearly affected. One of these horses was a Franches-Montagnes foal in a prospective cohort. It was carefully and repeatedly examined by clinical specialists until the age of 6 months without any signs of disease. A liver ultrasound at the age of 6 months was completely normal. The owners were asked to report any changes in the health status to us immediately. However, when we wanted to re-examine this foal at 12 months of age, the owners told us that they had it slaughtered in the meantime. We suspect that this horse was affected with a very late onset of disease or that disease was suppressed by hypothetical modifier gene(s). The remaining four discrepant horses were 3 adult Franches-Montagnes horses and one adult New Forest Pony, where the owners did not report any overt clinical signs. We speculate that liver disease in these horses has again been suppressed by hypothetical modifier gene(s).

Finally, we had one Franches-Montagnes foal that had the typical clinical symptoms of congenital hepatic fibrosis, but carried only one copy of the disease associated haplotype. We speculate that this horse might have acquired a second independent pathogenic mutation on the other *PKHD1* allele. However, unfortunately, the DNA of this animal was of insufficient quality for a whole genome sequencing experiment and therefore we are unable to confirm this hypothesis.

In conclusion, this study provides a naturally occurring large animal model for the liver-restricted subtype of human ARPKD. The horse phenotype is strongly associated with variants at the *PKHD1* gene. This study highlights that the identification of causative mutations for Mendelian traits remains a challenging task, if the underlying variants are located in non-coding regions or poorly annotated genes. The imperfect genotype-phenotype correlation possibly suggests the presence of additional modifier genes. The knowledge of disease linked SNPs allows indirect selection towards eradication of the disease from the Franches-Montagnes horse breed.

## Materials and Methods

### Ethics Statement

All animal work has been conducted according to national guidelines for animal welfare. The collection of the liver biopsy from a non-affected horse was approved by the canton of Bern (permit number BE114/12). The collection of blood samples from non affected horses was approved by the canton of Vaud (permit no. 2227). Blood samples from the CLF affected horses were taken for diagnostic purposes. Liver samples from CLF affected horses were taken post mortem. Thus the sample collection from CLF affected horses did not constitute an animal experiment and required no extra permit. All samples were taken with the owners’ consent or were voluntarily submitted by the owners for diagnostic purposes. CLF affected horses were either slaughtered (slaughterhouse Thun), euthanized due to humane reasons, or died as a result of their disease. No animal was sacrificed for this study.

The protocol for euthanasia of CLF affected foals involved sedation of the mare by intravenous injection of 1 mg/kg xylazine, if the foal was still with the mare. Subsequently, an intravenous catheter was placed into the jugular vein of the foal with lidocaine anesthesia at the placement site. The foal was then sedated by injection of 1 mg/kg xylazine into the catheter. Approximately 5 minutes after the sedation injection, the mare was separated from the foal and the CLF affected foal was euthanized by injection of 90 mg/kg pentobarbital into the catheter.

### Animal selection

A total of 2,123 Franches-Montagnes horses were investigated in this study. These included a total of 25 cases affected with congenital hepatic fibrosis and at least some DNA for genetic analysis available. The samples of the cases were collected over a period of 30 years (1984–2013) and include e.g. paraffin embedded formalin-fixed liver tissue samples animals from histopathological analyses of old cases.

Our cohort included 2,098 Franches-Montagnes horses that we considered as non-affected control horses. These included 173 healthy Franches-Montagnes older than 5 years with no report of liver disease until the time point of sampling. The other 1,925 Franches-Montagnes were either younger than 5 years, or no information about age or health status was available.

We additionally used 234 non-affected horses from genetically diverse horse breeds (Table S1, Table S2).

### DNA isolation and SNP genotyping

We isolated genomic DNA from EDTA blood and tissue samples with the Nucleon Bacc2 kit (GE Healthcare). The isolation of genomic DNA from paraffin embedded tissue samples was carried out using the DNeasyBlood & Tissue Kit (QIAGEN) combining the protocol for pretreatment for paraffin embedded tissue and the spin-column protocol for purification of total DNA from animal tissue as recommended by the manufacturer. The DNA of 5 recent cases with good DNA quality and 12 controls was genotyped by GeneSeek with the EquineSNP70 Genotyping BeadChip (Illumina) including 65,157 evenly distributed SNPs.

### Genome-wide association and homozygosity mapping

We used PLINK v1.07 [19] to perform genome-wide association analyses (GWAS). All 5 cases and 12 controls used for the analysis had call rates >90%. We removed markers with call rates <90% and with minor allele frequency (MAF)<5%. All remaining markers were in Hardy-Weinberg equilibrium (p>10^−5^). The final dataset consisted of 17 horses and 38,394 SNPs. We performed an allelic association study and determined an empirical significance threshold by performing 100,000 permutations of the dataset with arbitrarily assigned phenotypes.

We analyzed regions of shared homozygosity among the 5 cases on chromosome 20 by visual inspection of the genotypes in an Excel-file. We also performed a homozygosity search with PLINK v1.07 using the following parameters: –homozyg –homozyg-group –homozyg-kb 500 –homozyg-snp 25 –homozyg-match 0.95 –homozyg-het 1. The homozygous region on chromosome 20 was the only region in the entire genome that fulfilled our search criteria using these parameters.

### Whole genome re-sequencing

We prepared fragment libraries with a 200 bp–300 bp insert size and collected one lane of illumina HiSeq2000 paired-end reads (2×100 bp) per genome. Fastq files were created using Casava 1.8. We obtained a total of 234,536,171 (affected horse) and 568,567,625 (carrier) paired-end reads which were then mapped to the horse reference genome Broad/equCab2.0 and aligned using Burrows-Wheeler Aligner (BWA) version 0.5.9-r16 [20] with default settings. The mapping showed 204,062,157 and 517,388,289 reads had unique mapping positions revealing roughly a 7.55x and 19.1x coverage, respectively. The SAM file generated by BWA was then converted to BAM and the reads sorted by chromosome using samtools (http://samtools.sourceforge.net/). PCR duplicates were marked using Picard tools (http://sourceforge.net/projects/picard/). We used the Genome Analysis Tool Kit (GATK version 2.4.9, [21]) to perform local realignment and to produce cleaned BAM files. Variant calls were then made with the unified genotyper module of GATK. We also performed a visual inspection of the aligned reads in the integrative genomics viewer (IGV, [22]) to search for large structural variants. However, this analysis did not indicate any structural variation within the critical interval.

The variant data for each sample was obtained in variant call format (version 4.0) as raw calls for all samples and sites flagged using the variant filtration module of GATK. Variant filtration was done following best practice documentation of GATK version 4. The snpEFF software [23] together with the Broad/equCab2.0 Ensembl annotation version 2.69 was used to predict the functional effects of detected variants.

### RNA sequencing

We isolated total RNA from the liver of an affected and a non-affected horse and prepared two fragment libraries with 350 bp insert size following illumina’s TruSeq mRNA Sample Preparation Guide and collected half of a lane of illumina HiSeq2000 paired-end reads (2×100 bp) per library obtaining 152,265,046 (affected horse) and 121,033,908 tags (non-affected horse). We mapped the reads to the horse reference genome (Broad/equCab2.0) using the spliced alignment program TopHat2 with default parameters [24]. Read counting and differential gene expression analysis was performed using Cufflinks 2.0 software [25].

### RT-PCR

Using the same liver RNA samples described above we also amplified the *PKHD1* cDNA from position −52 to +10706 as a series of 9 overlapping RT-PCR products using SuperscriptII (LifeTechnologies) and AmpliTaqGold360 Mastermix (Life Technologies) according to the manufacturer’s recommendations. A transcript containing the complete equine *PKHD1* coding sequence was assembled from the RNA-seq data and Sanger sequences derived from the RT-PCR products (Figure S3).

### Sanger sequencing

The associated variants were genotyped by re-sequencing of targeted PCR products using Sanger sequencing technology. PCR products were amplified using AmpliTaqGold360 Mastermix (Life Technologies) and the products were directly sequenced on an ABI 3730 capillary sequencer (Life Technologies) after treatment with exonuclease I and shrimp alkaline phosphatase. Sequence data were analyzed with Sequencher 5.1 (GeneCodes).

## Supporting Information

Figure S1 **Alignment of the human PKHD1 protein (NP_619639.3) to the equine PKHD1 protein derived from a non-affected horse (translated from the sequence given in Figure S3).**
(DOCX)

Figure S2 **Conservation of the histidine residue at position 2038 and the isoleucine residue at position 2282 in the PKHD1 protein.**
(DOCX)

Figure S3 **Sequence of a predicted equine** ***PKHD1*** **transcript, which was assembled from Sanger-sequenced RT-PCR products and RNA-seq data.** This assembled mRNA sequence was derived from different horses and does not necessarily resemble one contiguous haplotype.
(TXT)

Table S1 **Horses used for whole genome sequencing.**
(DOCX)

Table S2 **Genotypes of 64 variants in the critical interval, which were homozygous variant in the sequenced genome of an affected Franches-Montagnes foal and either homozygous wildtype or heterozygous in the genome sequences of 29 non-affected Franches-Montagnes horses and 18 non-affected horses from other breeds.**
(DOCX)

Table S3 **Equine** ***PKHD1*** **annotation.**
(XLSX)
