# Supplementary material for: Congenital Hepatic Fibrosis in the Franches-Montagnes Horse Is Associated with the Polycystic Kidney and Hepatic Disease 1 (PKHD1) Gene
Source: PLoS One. 2014 Oct 8;9(10):e110125. doi: 10.1371/journal.pone.0110125 (PMC4190318; doi:10.1371/journal.pone.0110125)
Supplement: Figure S1 — Alignment of the human PKHD1 protein (NP_619639.3) to the equine PKHD1 protein derived from a non-affected horse (translated from the sequence given in Figure S3). (DOCX) [file pone.0110125.s001.docx]

**Figure S1**. Alignment of the human PKHD1 protein (NP_619639.3) to the equine PKHD1 protein derived from a non-affected horse (translated from Figure S3). Note the high overall sequence conservation with the exception of the region encoded by the human exon 42, which is missing from the equine transcript. The 14 identified missense variants in the equine sequence are highlighted with color. Variants that were not associated with the congenital hepatic fibrosis phenotype are highlighted in green, variants that were strongly, but not perfectly, associated with the disease are highlighted in red.

10 20 30 40 50 60

human MTAWLISLMSIEVLLLAVRHLSLHIEPEEGSLAGGTWITVIFDGLELGVLYPNNGSQLEI

::.:::::.:::.::::: .::.::::::::::::::::::::: . ..::: :::::::

horse MTTWLISLISIETLLLAVPYLSFHIEPEEGSLAGGTWITVIFDGSDSSLLYPANGSQLEI

10 20 30 40 50 60

70 80 90 100 110 120

human HLVNVNMVVPALRSVPCDVFPVFLDLPVVTCRTRSVLSEAHEGLYFLEAYFGGQLVSSPN

::... .:::: :.:::: ::::::::: :::::. ::::::::.: :. ::..:: .

horse HLMSA--AVPALPSIPCDVVPVFLDLPVVMCRTRSLPSEAHEGLYYLGAHSQGQVASSST

70 80 90 100 110

E109G

130 140 150 160 170 180

human PGPRDSCTFKFSKAQTPIVHQVYPPSGVPGKLIHVYGWIITGRLETFDFDAEYIDSPVIL

:::.:: :::::.::::.:.:: :::::::.::.::::::: : :::::::::::::.::

horse PGPQDSPTFKFSRAQTPFVYQVNPPSGVPGELIQVYGWIITRRSETFDFDAEYIDSPLIL

120 130 140 150 160 170

190 200 210 220 230 240

human EAQGDKWVTPCSLINRQMGSCYPIQEDHGLGTLQCHVEGDYIGSQNVSFSVFNKGKSMVH

:::::.:::::::.::: :: ::.:: ::::::::.::: ::::::::::::::::::::

horse EAQGDRWVTPCSLVNRQTGSRYPVQEHHGLGTLQCRVEGHYIGSQNVSFSVFNKGKSMVH

180 190 200 210 220 230

250 260 270 280 290 300

human KKAWLISAKQDLFLYQTHSEILSVFPETGSLGGRTNITITGDFFDNSAQVTIAGIPCDIR

:.::::::.::::::::.:::::: ::::::::::.::::: :::: :::::::::::::

horse KNAWLISANQDLFLYQTYSEILSVVPETGSLGGRTDITITGAFFDNPAQVTIAGIPCDIR

240 250 260 270 280 290

310 320 330 340 350 360

human HVSPRKIECTTRAPGKDVRLTTPQPGNRGLLFEVGDAVEGLELTEATPGYRWQIVPNASS

::::::: :::::::: .:::.:: ::::::::::::.:::.::::::::::::::::::

horse HVSPRKIACTTRAPGKGTRLTAPQAGNRGLLFEVGDAAEGLDLTEATPGYRWQIVPNASS

300 310 320 330 340 350

370 380 390 400 410 420

human PFGFWSQEGQPFRARLSGFFVAPETNNYTFWIQADSQASLHFSWSEEPRTKVKVASISVG

::::::.:::::::::::::::::::::::::::::::.:.:: ::.:::::::::: ::

horse PFGFWSKEGQPFRARLSGFFVAPETNNYTFWIQADSQATLYFSQSEDPRTKVKVASIRVG

360 370 380 390 400 410

430 440 450 460 470 480

human TADWFDSWEQNRDEGTWQQKTPKLELLGGAMYYLEAEHHGIAPSRGMRIGVQIHNTWLNP

::::::::::. .:::::::::::::.::: ::::::::: :::::::::::::::::::

horse TADWFDSWEQKGNEGTWQQKTPKLELFGGARYYLEAEHHGRAPSRGMRIGVQIHNTWLNP

420 430 440 450 460 470

490 500 510 520 530 540

human DVVTTYLREKHQIRVRAQRLPEVQVLNVSGRGNFFLTWDNVSSQPIPANATAHLIQTTIE

:::.:::.::::::.:::::::.:.:.:::::::.:::::::::::::::::: :::..:

horse DVVSTYLQEKHQIRIRAQRLPEIQMLTVSGRGNFLLTWDNVSSQPIPANATAHQIQTALE

480 490 500 510 520 530

550 560 570 580 590 600

human ELLAVKCKLEPLWSNILLRLGFERGPEVSNSDGDLTSGTEPFCGRFSLRQPRHLVLTPPA

:::::::::::: .:::..::::.::: :.:::::::::::::::::: :::::::::::

horse ELLAVKCKLEPLSANILFQLGFEEGPEGSSSDGDLTSGTEPFCGRFSLYQPRHLVLTPPA

540 550 560 570 580 590

R557Q

610 620 630 640 650 660

human AQKGYRLDQYTHLCLAYKGHMNKILKMIVSFTIGFQNMVKNTTCDWSLTRTSPESWQFDC

:::::.::::::::.::::::: :::. ::::. :::..:: :::: : :::.:::: :

horse AQKGYQLDQYTHLCMAYKGHMNTILKVTVSFTVDFQNVAKNITCDWRLEGTSPNSWQFTC

600 610 620 630 640 650

R604Q

670 680 690 700 710 720

human TDLWETCVRCFGDLQPPPANSPVLVHQINLLPLAQETGLFYVDEIIIADTNVTVSQADSG

::::.:::. ::::: :.::.::: :.::::.:: :.::.:::.:::::.::::::::

horse TDLWNTCVHRSTDLQPPLASSPALVHLIDLLPLSQEPGVFYMDEIVIADTNLTVSQADSG

660 670 680 690 700 710

730 740 750 760 770 780

human TARPGGNLVESVSVVGSPPVYSVTSWLAGCGTELPLITARSVPTEGTEEGSGLVLVTTQR

:::::::::: .:::::::::.:: :::::: :::::.: ::::::.:: :::: :::::

horse TARPGGNLVELLSVVGSPPVYNVTCWLAGCGPELPLISASSVPTEGAEERSGLVHVTTQR

720 730 740 750 760 770

790 800 810 820 830 840

human RQRTSPPLGGHFRIQLPNTVISDVPVQISAHHLHQLLQNNADDFTSRYLNASDFTVKEDL

:::: :::::::::: :::: ::::.::: :::.::.:::::::.::::.:::.: :::

horse LQRTSLPLGGHFRIQLSNTVIPDVPVHISASHLHKLLRNNADDFTARYLNVSDFSVMEDL

780 790 800 810 820 830

850 860 870 880 890 900

human YTCYEHVWTLSWSTQIGDLPNFIRVSDENLTGVNPAAATRVVYDGGVFLGPIFGDMLATA

.:::.:::::::.:.:::::::::::::::::::.:.:::::::::::::::::::.::

horse KSCYERVWTLSWSAQVGDLPNFIRVSDENLTGVNPVATTRVVYDGGVFLGPIFGDMLVTA

840 850 860 870 880 890

910 920 930 940 950 960

human NQHTQVVVRVNDVPAHCPGSCSFQYLQGSTPCVHSVWYSIDGDINLMIYITGTGFSGDSQ

:. ::::::::.:::: :::::.::..::: :::: :: ::: .:..:::::.:::: .

horse NRCPQVVVRVNDIPAHCSGSCSFRYLEASTPRVHSVCYSPDGDTDLLVYITGTSFSGDYK

900 910 920 930 940 950

970 980 990 1000 1010 1020

human FLQVTVNKTSCKVIFSNQTNVVCQTDLLPVGMHRILMLVRPSGLAISATGEDLFLNVKPR

:::::::::::::::::::::::.::::::.:.: ::::::: ::.:.: :::::.::

horse ALQVTVNKTSCKVIFSNQTNVVCQADLLPVGVHQISMLVRPSGRAINASGGGLFLNVEPR

960 970 980 990 1000 1010

1030 1040 1050 1060 1070 1080

human LDMVEPSRAADIGGLWATIRGSSLEGVSLILFGSYSCAINVATSNSSRIQCKVPPRGKDG

:: :::::::.:::::::::::::: :::.:::: ::.:::.:::: :::::::::::::

horse LDAVEPSRAAEIGGLWATIRGSSLEDVSLVLFGSQSCVINVTTSNSRRIQCKVPPRGKDG

1020 1030 1040 1050 1060 1070

1090 1100 1110 1120 1130 1140

human RIVNVTVIRGDYSAVLPRAFTYVSSLNPVIVTLSRNISNIAGGETLVIGVARLMNYTDLD

..::::::: :.:.::: :::: :::::::..:::: :.::::::: ::.: :.: ::::

horse HVVNVTVIREDHSTVLPMAFTYDSSLNPVITSLSRNRSSIAGGETLFIGMALLVNDTDLD

1080 1090 1100 1110 1120 1130

1150 1160 1170 1180 1190 1200

human VEVHVQDALAPVHTQSAWGLEVALPPLPAGLHRISVSINGVSIHSQGVDLHIQYLTEVFS

:.::.:..::::: : : ::::.:: :::::::::::::::.: ::::::::. .:::::

horse VQVHIQETLAPVHEQMAQGLEVVLPLLPAGLHRISVSINGVNISSQGVDLHIHCITEVFS

1140 1150 1160 1170 1180 1190

1210 1220 1230 1240 1250 1260

human IEPCCGSLLGGTILSISGIGFSRDPALVWVLVGNRSCDIVNLTEASIWCETLPAPQIPDA

::::::::::::::::::::::::::::::::::.:::::: :: .::::: :: .:::

horse IEPCCGSLLGGTILSISGIGFSRDPALVWVLVGNQSCDIVNSTERNIWCETSPASLLPDA

1200 1210 1220 1230 1240 1250

E1242K

1270 1280 1290 1300 1310 1320

human GAPTVPAAVEVWAGNRFFARGPSPSLVGKGFTFMYEAAATPVVTAMQGEITNSSLSLHVG

.::: ::::::. . .::::::::::: ::::.::::::::..::::.::: : :

horse DDLSVPAPVEVWAGSTSIPQGPSPSLVGKGFIFMYEVAATPVVTAVRGEITDSSLRLDVE

1260 1270 1280 1290 1300 1310

S1277P; R1278Q; A1279G

1330 1340 1350 1360 1370 1380

human GSNLSNSVILLGNLNCDVETQSFQGNVSLSGCSIPLHSLEAGIYPLQVRQKQMGFANMSV

::::::::::::.: : .:::::..::::::::.:::::::::::::::::::::::::.

horse GSNLSNSVILLGGLACGLETQSFRSNVSLSGCSFPLHSLEAGIYPLQVRQKQMGFANMSA

1320 1330 1340 1350 1360 1370

1390 1400 1410 1420 1430 1440

human VLQQFAVMPRIMAIFPSQGSACGGTILTVRGLLLNSRRRSVRVDLSGPFTCVILSLGDHT

: :::.: ::: ::::..:::::::.:::.:: :.::: ::.:.::::::::::::::.:

horse VPQQFVVTPRITAIFPAHGSACGGTVLTVQGLALSSRRGSVQVNLSGPFTCVILSLGDQT

1380 1390 1400 1410 1420 1430

D1422N

1450 1460 1470 1480 1490 1500

human ILCQVSLEGDPLPGASFSLNVTVLVNGLTSECQGNCTLFIREEASPVMDALSTNTSGSLT

.:::. : :::::::::.:::::::: : :::::.::::.:::..::.:::.:: :::::

horse VLCQIHLVGDPLPGASFTLNVTVLVNELPSECQGDCTLFLREETTPVVDALTTNISGSLT

1440 1450 1460 1470 1480 1490

A1488S

1510 1520 1530 1540 1550 1560

human TVLIRGQRLATTADEPMVFVDDQLPCNVTFFNASHVVCQTRDLAPGPHYLSVFYTRNGYA

:::::::::.::::::.: :::.: :::::::::::.: :.:::::::::. :::::

horse TVLIRGQRLGTTADEPVVSVDDHLLCNVTFFNASHVTCWISGLTPGPHYLSVFHRRNGYA

1500 1510 1520 1530 1540 1550

1570 1580 1590 1600 1610 1620

human CSGNVSRHFYIMPQVFHYFPKNFSLHGGSLLTIEGTGLRGQNTTSVYIDQQTCLTVNIGA

::::::::: :.::::.:::::::.:::.:::.:::.:::::.: ::. :.:::::...

horse CSGNVSRHFDILPQVFRYFPKNFSIHGGGLLTVEGTALRGQNATLVYVGWQACLTVNVSS

1560 1570 1580 1590 1600 1610

1630 1640 1650 1660 1670 1680

human ELIRCIVPTGNGSVALEIEVDGLWYHIGVIGYNKAFTPELISISQSDDILTFAVAQISGA

.::.::::.:::::::.:::: : ...:::.:...:::::.:.::.::.:::::::::::

horse DLIQCIVPSGNGSVALNIEVDRLSHQMGVISYSNTFTPELLSLSQTDDVLTFAVAQISGA

1620 1630 1640 1650 1660 1670

1690 1700 1710 1720 1730 1740

human ANIDIFIGMSPCVGVSGNHTVLQCVVPSLPAGEYHVRGYDCIRGWASSALVFTSRVIITA

:.::.::::::..::::.:::::::::::::::.::::: :::::::::::::: .::

horse MNVDILIGMSPCMNVSGNRTVLQCVVPSLPAGEYQVRGYDRTRGWASSALVFTSRVSVTA

1680 1690 1700 1710 1720 1730

1750 1760 1770 1780 1790 1800

human VTENFGCLGGRLVHVFGAGFSPGNVSAAVCGAPCRVLANATVSAFSCLVLPLDVSLAFLC

::.:::::::::::: :::: : :.:::::::::.:::::::::::::::::::::::::

horse VTQNFGCLGGRLVHVSGAGFPPENISAAVCGAPCQVLANATVSAFSCLVLPLDVSLAFLC

1740 1750 1760 1770 1780 1790

1810 1820 1830 1840 1850 1860

human GLKREEDSCEAARHTYVQCDLTVAMATEQLLESWPYLYICEESSQCLFVPDHWAESMFPS

:::.::..:.:. .:::::::::...::.:: ::::.:::::: .:::.: ::.:: :

horse GLKHEEEGCDASSRTYVQCDLTVTVGTESLLSSWPYFYICEESPSCLFAPGHWTESASPW

1800 1810 1820 1830 1840 1850

1870 1880 1890 1900 1910 1920

human FSGLFISPKLERDEVLIYNSSCNITMETEAEMECETPNQPITVKITEIRKRWGQNTQGNF

:::::::::.::::::::::::::::::::.:::::::::::.::::::. .:::::::

horse FSGLFISPKVERDEVLIYNSSCNITMETEAKMECETPNQPITAKITEIRESRAQNTQGNF

1860 1870 1880 1890 1900 1910

1930 1940 1950 1960 1970 1980

human SLQFCRRWSRTHSWFPERLPQDGDNVTVENGQLLLLDTNTSILNLLHIKGGKLIFMAPGP

:.::::::::.:::::::.::::::::::.:::::::::::::::::.:::::::: :::

horse SFQFCRRWSRAHSWFPERVPQDGDNVTVEKGQLLLLDTNTSILNLLHVKGGKLIFMDPGP

1920 1930 1940 1950 1960 1970

1990 2000 2010 2020 2030 2040

human IELRAHAILVSDGGELRIGSEDKPFQGRAQITLYGSSYSTPFFPYGVKFLAVRNGTLSLH

::::::.:::: ::::::::.::::::.:.: :::::.::::::::::::::::::::::

horse IELRAHSILVSHGGELRIGSKDKPFQGKAEIKLYGSSHSTPFFPYGVKFLAVRNGTLSLH

1980 1990 2000 2010 2020 2030

K1999E H2038Y

2050 2060 2070 2080 2090 2100

human GSLPEVIVTCLRATAHALDTVLALEDAVDWNPGDEVVIISGTGVKGAKPMEEIVTVETVQ

: ::::.:: :::.:.: ::::::::::::.:::::::::: :: ::::::::: ::::.

horse GLLPEVMVTHLRAAAYARDTVLALEDAVDWHPGDEVVIISGIGVAGAKPMEEIVIVETVH

2040 2050 2060 2070 2080 2090

2110 2120 2130 2140 2150 2160

human DTDLYLKSPLRYSHNFTENWVAGEHHILKATVALLSRSITIQGNLTNEREKLLVSCQEAN

..::.:.::::::::::::::::::::::. :.::::.:::.::::::: :::.:::::.

horse NADLHLRSPLRYSHNFTENWVAGEHHILKVMVVLLSRNITIRGNLTNERMKLLASCQEAS

2100 2110 2120 2130 2140 2150

2170 2180 2190 2200 2210 2220

human APEGNLQHCLYSMSEKMLGSRDMGARVIVQSFPEEPSQVQLKGVQFQVLGQAFHKHLSSL

: :::::.:::: :::::::::.:::.::::.: :::.:::::: :. :::::.::::::

horse ASEGNLQNCLYSKSEKMLGSRDLGARLIVQSLPGEPSRVQLKGVLFRELGQAFRKHLSSL

2160 2170 2180 2190 2200 2210

2230 2240 2250 2260 2270 2280

human TLVGAMRESFIQGCTVRNSFSRGLSMCGTLGLKVDSNVFYNILGHALLVGTCTEMRYISW

:::::::.:..::::: .:::::::: :::::: ::::::::::::::

horse TLVGAMRDSYLQGCTVWGSFSRGLSMSRTLGLKVTSNVFYNILGHALLV-----------

2220 2230 2240 2250 2260

2290 2300 2310 2320 2330 2340

human EAIHGRKDDWSGHGNIIRNNVIIQVSGAEGLSNPEMLTPSGIYICSPTNVIEGNRVCGAG

: : .:.::::::::.::::::::.::.:::::::: .::::.::::::.::

horse --------DESEQGSIIRNNVIIRVSGAEGLSSPEVLTPSGIYIRNPTNVVEGNRVCAAG

2270 2280 2290 2300 2310

I2282N

2350 2360 2370 2380 2390 2400

human YGYFFHLMTNQTSQAPLLSFTQNIAHSCTRYGLFVYPKFQPPWDNVTGTTLFQSFTVWES

:::::::.:..:::::::::: :.::::::::::::::::::::. :: ::.:.: :: .

horse YGYFFHLVTSRTSQAPLLSFTGNVAHSCTRYGLFVYPKFQPPWDDGTGPTLIQNFMVWGG

2320 2330 2340 2350 2360 2370

2410 2420 2430 2440 2450 2460

human AGGAQIFRSSNLRLKNFKVYSCRDFGIDVLESDANTSVTDSLLLGHFAHKGSLCMSSGIK

:::::::::::: ::::..:::::::::.:::::::::::::::::::::::::::.:::

horse AGGAQIFRSSNLLLKNFQIYSCRDFGIDILESDANTSVTDSLLLGHFAHKGSLCMSAGIK

2380 2390 2400 2410 2420 2430

2470 2480 2490 2500 2510 2520

human TPKRWELMVSNTTFVNFDLINCVAIRTCSDCSQGQGGFTVKTSQLKFTNSSNLVAFPFPH

:::: ::.::::::::::: .:::::::: ::.:::::::::.::::::: ::::::: :

horse TPKRQELVVSNTTFVNFDLTECVAIRTCSGCSRGQGGFTVKTNQLKFTNSPNLVAFPFAH

2440 2450 2460 2470 2480 2490

2530 2540 2550 2560 2570 2580

human AAILEDLDGSLSGKNRSHILASMETLSASCLVNSSFGRVVHGSACGGGVLFHRMSIGLAN

:::::::::::::::::::::::::::::: ::.::..:: ::.:: ::::::::::::

horse AAILEDLDGSLSGKNRSHILASMETLSASCWVNTSFSQVVSGSVCGEDVLFHRMSIGLAN

2500 2510 2520 2530 2540 2550

2590 2600 2610 2620 2630 2640

human TPEVSYDLTMTDSRNKTTTVNYVRDTLSNPRGWMALLLDQETYSLQSENLWINRSLQYSA

.:.:: ::::::::::::::::::::::: :::::::::: :::. :. ::.:::::::

horse APDVSSDLTMTDSRNKTTTVNYVRDTLSNRYGWMALLLDQEMYSLRFETPWISRSLQYSA

2560 2570 2580 2590 2600 2610

2650 2660 2670 2680 2690 2700

human TFDNFAPGNYLLLVHTDLPPYPDILLRCGSRVGLSFPFLPSPGQNQGCDWFFNSQLRQLT

:::::::::::::::.:. ::::::. :::.:: :.: :::::..:::::::.:::::::

horse TFDNFAPGNYLLLVHADVWPYPDILMWCGSHVGRSLPSLPSPGRDQGCDWFFDSQLRQLT

2620 2630 2640 2650 2660 2670

2710 2720 2730 2740 2750 2760

human YLVSGEGQVQVILRVKEGMPPTISASTSAPESALKWSLPETWQGVEEGWGGYNNTIPGPG

:::::::::.:::.::::.:::::::::.:::::::: ::.: ::::::::.:.: ::::

horse YLVSGEGQVRVILQVKEGVPPTISASTSVPESALKWSHPEAWTGVEEGWGGHNHTTPGPG

2680 2690 2700 2710 2720 2730

2770 2780 2790 2800 2810 2820

human DDVLILPNRTVLVDTDLPFFKGLYVMGTLDFPVDRSNVLSVACMVIAGGELKVGTLENPL

:::::::::::::::::::.:::::::::.:::::::.::::::::::::::::::::::

horse DDVLILPNRTVLVDTDLPFLKGLYVMGTLEFPVDRSNILSVACMVIAGGELKVGTLENPL

2740 2750 2760 2770 2780 2790

2830 2840 2850 2860 2870 2880

human EKEQKLLILLRASEGVFCDRMNGIHIDPGTIGVYGKVHLYSAYPKNSWTHLGADIASGNE

::::::::::::::::::::..:::::::::::::::.:.:::::.::::::::::::::

horse EKEQKLLILLRASEGVFCDRFDGIHIDPGTIGVYGKVQLHSAYPKKSWTHLGADIASGNE

2800 2810 2820 2830 2840 2850

2890 2900 2910 2920 2930 2940

human RIIVEDAVDWRPHDKIVLSSSSYEPHEAEVLTVKEVKGHHVRIYERLKHRHIGSVHVTED

:::::::::::::::::::::::::::.::::::::::::::..:::::::::.::: ::

horse RIIVEDAVDWRPHDKIVLSSSSYEPHETEVLTVKEVKGHHVRLHERLKHRHIGNVHVMED

2860 2870 2880 2890 2900 2910

2950 2960 2970 2980 2990 3000

human GRHIRLAAEVGLLTRNIQIQPDVSCRGRLFVGSFRKSSREEFSGVLQLLNVEIQNFGSPL

::.::::::::::::::::: :.::.::: :::::::::::::::::: :::::::::::

horse GRYIRLAAEVGLLTRNIQIQSDTSCKGRLRVGSFRKSSREEFSGVLQLSNVEIQNFGSPL

2920 2930 2940 2950 2960 2970

3010 3020 3030 3040 3050 3060

human YSSVEFSNVSAGSWIISSTLHQSCGGGIHAAASHGVLLNDNIVFGTAGHGIDLEGQAYTV

:::.:..:::::::::::::::::.:::::.::::..:::::::::::::::::::::..

horse YSSIELTNVSAGSWIISSTLHQSCSGGIHAVASHGIILNDNIVFGTAGHGIDLEGQAYSL

2980 2990 3000 3010 3020 3030

3070 3080 3090 3100 3110 3120

human TNNLVVLMTQPAWSTIWVAGIKVNQVKDINLHGNVVAGSERLGFHIRGHKCSSCELLWSD

.::::::::: ::::.::::::::..:::::.:::::::::::::::::.::: : ::::

horse SNNLVVLMTQSAWSTVWVAGIKVNRAKDINLRGNVVAGSERLGFHIRGHSCSSPEALWSD

3040 3050 3060 3070 3080 3090

3130 3140 3150 3160 3170 3180

human NVAHSSLHGLHLYKESGLDNCTRISGFLAFKNFDYGAMLHVENSVEIENITLVDNTIGLL

:::::::::::.:.:::::::: :::::::::::::::::::::::.::::::::..:::

horse NVAHSSLHGLHFYQESGLDNCTGISGFLAFKNFDYGAMLHVENSVEMENITLVDNAVGLL

3100 3110 3120 3130 3140 3150

3190 3200 3210 3220 3230 3240

human AVVYVFSAPQNSVKKVQIVLRNSVIVATSSSFDCIQDKVKPHSANLTSTDRAPSNPRGGR

::::: :.::.:. .::::::::::::::::::::::..:: ::: ::::::::::.:::

horse AVVYVSSVPQSSIGNVQIVLRNSVIVATSSSFDCIQDRIKPCSANSTSTDRAPSNPKGGR

3160 3170 3180 3190 3200 3210

3250 3260 3270 3280 3290 3300

human IGILWPVFTSEPNQWPQEPWHKVRNDHSISGIMKLQDVTFSSFVKSCYSDDLDVCILPNA

::::::.:::::::::::::::::: ::.:::::::::::::::::::::::::::::::

horse IGILWPAFTSEPNQWPQEPWHKVRNGHSVSGIMKLQDVTFSSFVKSCYSDDLDVCILPNA

3220 3230 3240 3250 3260 3270

3310 3320 3330 3340 3350 3360

human ENSGIMHPITAERTRMLKIKDKNKFYFPSLQPRKDLGKVVCPELDCASPRKYLFKDLDGR

:: ::::::::::::::::::::::::: :: ::::: .::::::: :::::::::::::

horse ENVGIMHPITAERTRMLKIKDKNKFYFPPLQTRKDLGILVCPELDCESPRKYLFKDLDGR

3280 3290 3300 3310 3320 3330

3370 3380 3390 3400 3410 3420

human ALGLPPPVSVFPKTEAEWTASFFNAGTFREEQKCTYQFLMQGFICKQTDQVVLILDSADA

::::::::::::: :::::.::::.:::::::::.:. :.::.::::::::.::::.:::

horse ALGLPPPVSVFPKIEAEWTGSFFNTGTFREEQKCVYRPLIQGYICKQTDQVILILDNADA

3340 3350 3360 3370 3380 3390

3430 3440 3450 3460 3470 3480

human IWAIQKLYPVVSVTSGFVDVFSSVNANIPCSTSGSVSTFYSILPIRQITKVCFMDQTPQV

::..::::::::::::::.::::::: :::::::::::::::: :.::::::.::::::

horse TWAMRKLYPVVSVTSGFVDTFSSVNANTPCSTSGSVSTFYSILPTREITKVCFVDQTPQV

3400 3410 3420 3430 3440 3450

3490 3500 3510 3520 3530 3540

human LRFFLLGNKSTSKLLLAVFYHELQSPHVFLGESFIPPTLVQSASLLLNESIGANYFNIMD

::::::::::::::::::::::::::.:::: ::::::::::.: ::.: ::.:::.:::

horse LRFFLLGNKSTSKLLLAVFYHELQSPQVFLGGSFIPPTLVQSTSSLLDEPIGSNYFSIMD

3460 3470 3480 3490 3500 3510

3550 3560 3570 3580 3590 3600

human NLLYVVLQGEEPIEIRSGVSIHLALTVMVSVLEKGWEIVILERLTNFLQIGQNQIRFIHE

::::::::::::.:: : .:.::.:::: :.::::::....::::.::::::.:::: ::

horse NLLYVVLQGEEPVEILSDASLHLVLTVMFSILEKGWEVMFFERLTEFLQIGQDQIRFTHE

3520 3530 3540 3550 3560 3570

L3535R

3610 3620 3630 3640 3650 3660

human MPGHEETLKAIADSRAKRKRNCPTVTCTSHYRRVGQRRPLMMEMNSHRASPPMTVETISK

:::.: :::::::::.:::::::::::..::: .:.:::::.::.:::. :: :.: :::

horse MPGNEATLKAIADSRTKRKRNCPTVTCANHYR-AGRRRPLMIEMSSHRVPPPTTTEPISK

3580 3590 3600 3610 3620 3630

R3599H

3670 3680 3690 3700 3710 3720

human VIVIEIGDSPTVRSTGMISSLSSNKLQNLAHRVITAQQTGVLENVLNMTIGALLVTQSKG

:.:::::: :::.::: : ::::::::::::..:::::::.::::::::::.:.::::::

horse VMVIEIGDLPTVKSTGPIPSLSSNKLQNLAHQIITAQQTGLLENVLNMTIGGLMVTQSKG

3640 3650 3660 3670 3680 3690

3730 3740 3750 3760 3770 3780

human VIGYGNTSSFKTGNLIYIRPYALSILVQPSDGEVGNELPVQPQLVFLDEQNRRVESLGPP

::::::::::::::::::::::::.::::::::::.::::::::::::.::::::::: :

horse VIGYGNTSSFKTGNLIYIRPYALSVLVQPSDGEVGKELPVQPQLVFLDKQNRRVESLGTP

3700 3710 3720 3730 3740 3750

3790 3800 3810 3820 3830 3840

human SEPWTISASLEGASDSVLKGCTQAETQDGYVSFYNLAVLISGSNWHFIFTVTSPPGVNFT

::::..: ::::.::::::::::::.::::: : ::::::::::::::::: ::::::::

horse SEPWAVSISLEGTSDSVLKGCTQAEAQDGYVRFSNLAVLISGSNWHFIFTVISPPGVNFT

3760 3770 3780 3790 3800 3810

3850 3860 3870 3880 3890 3900

human ARSKPFAVLPVTRKEKSTIILAASLSSVASWLALSCLVCCWLKRSKSRKTKPEEIPESQT

:::.:::.:::::.:.::::::::: ::.::::: ::::::.:.::::: : ::: ::::

horse ARSRPFAILPVTRSESSTIILAASLCSVVSWLALCCLVCCWFKKSKSRKIKSEEISESQT

3820 3830 3840 3850 3860 3870

3910 3920 3930 3940 3950

human NNQNIHIHISSKRRESQGPK-KEDTVVGEDMRMKVMLGKVNQCPHQLMNGVSRRKVSRHI

:.:. :. :::. :: :::.:.::::: ::::::.:: ::: .::::::::::.

horse NDQKNPTHVPSKRQGSQVETGKEDAVMGEDMRKKVMLGKLNQLPHQSLNGVSRRKVSRRT

3880 3890 3900 3910 3920 3930

3960 3970 3980 3990 4000 4010

human VREEE-----AAVPAPGTTGITSHGHICAPGAPAQQVYLQETGNWKEGQEQLLRYQLAGQ

: ::. ::::::. :..::::: ::::.::.:: .:::::::..::::::::::::

horse VGEEHGSQEGAAVPAPNLTSLTSHGHTCAPGSPARQVCVQETGNWKKAQEQLLRYQLAGQ

3940 3950 3960 3970 3980 3990

4020 4030 4040 4050 4060 4070

human NQLLLLCPDFRQERQQLPGQSRLSKQSGSLGLSQEKKASCGATEAFCLHSVHPETIQEQL 4074

.::::::::.:::::.. :::.:.:..: ::::::::.::::::.::::::.::::::::

horse DQLLLLCPDLRQERQRMQGQSQLGKEGGRLGLSQEKKTSCGATESFCLHSVRPETIQEQL 4058

4000 4010 4020 4030 4040 4050
