# Supplementary material for: Congenital Hepatic Fibrosis in the Franches-Montagnes Horse Is Associated with the Polycystic Kidney and Hepatic Disease 1 (PKHD1) Gene
Source: PLoS One. 2014 Oct 8;9(10):e110125. doi: 10.1371/journal.pone.0110125 (PMC4190318; doi:10.1371/journal.pone.0110125)
Supplement: Figure S2 — Conservation of the histidine residue at position 2038 and the isoleucine residue at position 2282 in the PKHD1 protein. (DOCX) [file pone.0110125.s002.docx]

**Figure S2**. Conservation of the histidine residue at position 2038 and the isoleucine residue at position 2282 in the PKHD1 protein. The sequences were derived from the following database accessions: *H. sapiens* NP_619639.3, *P. troglodytes* XP_518534.2, *B. taurus* XP_002697357.1, *C. lupus* XP_532169.2, *M. musculus* NP_694819.2, and *G. gallus* XP_420050.3.

p.H2038Y

horse LAVRNGTLSL**H**GLLPEVMVTH

human ............S....I..C

chimpanzee ............S....I..R

cattle ............S....V...

dog .................TF..

mouse ............SV...T..Y

chicken .........I..WV.K.TF.Y

p.I2282N

horse QGSIIRNNVI**I**RVSGAEGLSS

human H.N.......**.**Q........N

chimpanzee H.N.......**.**.........N

cattle ..N.......**.**..........

dog ..N.......**.**SI..T.....

mouse ...T......**.**S..A.....G

chicken K.NR....TV**.**GL.ATD...N
