# Supplementary material for: Congenital Hepatic Fibrosis in the Franches-Montagnes Horse Is Associated with the Polycystic Kidney and Hepatic Disease 1 (PKHD1) Gene
Source: PLoS One. 2014 Oct 8;9(10):e110125. doi: 10.1371/journal.pone.0110125 (PMC4190318; doi:10.1371/journal.pone.0110125)
Supplement: Table S1 — Horses used for whole genome sequencing. (DOCX) [file pone.0110125.s004.docx]

**Table S1.** Horses used for whole genome sequencing.

| Horse Breed | Number of animals |
| --- | --- |
| Franches-Montagnes | 30 |
| German Warmblood | 14 |
| Haflinger | 1 |
| Swiss Warmblood | 3 |
| UK Warmblood | 2 |
