# Supplementary material for: Congenital Hepatic Fibrosis in the Franches-Montagnes Horse Is Associated with the Polycystic Kidney and Hepatic Disease 1 (PKHD1) Gene
Source: PLoS One. 2014 Oct 8;9(10):e110125. doi: 10.1371/journal.pone.0110125 (PMC4190318; doi:10.1371/journal.pone.0110125)
Supplement: Table S2 — Genotypes of 64 variants in the critical interval, which were homozygous variant in the sequenced genome of an affected Franches-Montagnes foal and either homozygous wildtype or heterozygous in the genome sequences of 29 non-affected Franches-Montagnes horses and 18 non-affected horses from other breeds. (DOCX) [file pone.0110125.s005.docx]

**Table S2.** Genotypes of 64 variants in the critical interval, which were homozygous variant in the sequenced genome of an affected Franches-Montagnes foal and either homozygous wildtype or heterozygous in the genome sequences of 29 non-affected Franches-Montagnes horses and 18 non-affected horses from other breeds.

| **Chromosome 20 variant** | **Position & effect on amino acid sequence** | **Franches-Montagnes cases** | **Franches-Montagnes**  **controls** | **Horses from**  **other breeds^a^** |
| --- | --- | --- | --- | --- |
|  |  |  |  |  |
| g,49,167,150T>G | intergenic |  |  |  |
| *T/T* |  | - | 27 | 16 |
| *G/T* |  | - | 2 | 2 |
| *G/G* |  | 1 | - | - |
| g,49,198,172C>T | intergenic |  |  |  |
| *C/C* |  | - | 18 | 13 |
| *C/T* |  | - | 11 | 5 |
| *T/T* |  | 1 | - | - |
| g,49,237,104G>C | intergenic |  |  |  |
| *G/G* |  | - | 19 | 11 |
| *C/G* |  | - | 10 | 7 |
| *C/C* |  | 1 | - | - |
| g,49,238,942C>T | intergenic |  |  |  |
| *C/C* |  | - | 19 | 11 |
| *C/T* |  | - | 10 | 7 |
| *T/T* |  | 1 | - | - |
| g,49,239,468A>G | intergenic |  |  |  |
| *A/A* |  | - | 19 | 13 |
| *A/G* |  | - | 10 | 5 |
| *G/G* |  | 1 | - | - |
| g,49,239,766A>G | intergenic |  |  |  |
| *A/A* |  | - | 19 | 13 |
| *A/G* |  | - | 10 | 5 |
| *G/G* |  | 1 | - | - |
| g,49,244,857G>A | intergenic |  |  |  |
| *G/G* |  | - | 19 | 13 |
| *A/G* |  | - | 10 | 5 |
| *A/A* |  | 1 | - | - |
| g,49,245,184A>G | intergenic |  |  |  |
| *A/A* |  | - | 15 | 11 |
| *A/G* |  | - | 14 | 7 |
| *G/G* |  | 1 | - | - |
| g,49,245,187A>G | intergenic |  |  |  |
| *A/A* |  | - | 15 | 11 |
| *A/G* |  | - | 14 | 7 |
| *G/G* |  | 1 | - | - |
| g,49,248,035A>G | intergenic |  |  |  |
| *A/A* |  | - | 18 | 9 |
| *A/G* |  | - | 11 | 9 |
| *G/G* |  | 1 | - | - |
| g,49,252,551T>C | intergenic |  |  |  |
| *T/T* |  | - | 20 | 13 |
| *C/T* |  | - | 9 | 5 |
| *C/C* |  | 1 | - | - |
| g,49,255,288A>G | intergenic |  |  |  |
| *A/A* |  | - | 20 | 11 |
| *A/G* |  | - | 9 | 7 |
| *G/G* |  | 1 | - | - |
| g,49,263,717G>A | intergenic |  |  |  |
| *G/G* |  | - | 20 | 11 |
| *A/G* |  | - | 9 | 7 |
| *A/A* |  | 1 | - | - |
| g,49,278,254A>C | intergenic |  |  |  |
| *A/A* |  | - | 20 | 13 |
| *A/C* |  | - | 9 | 5 |
| *C/C* |  | 1 | - | - |
| g,49,283,407A>G | intergenic |  |  |  |
| *A/A* |  | - | 19 | 14 |
| *A/G* |  | - | 10 | 4 |
| *G/G* |  | 1 | - | - |
| g,49,304,637T>C | intergenic |  |  |  |
| *T/T* |  | - | 17 | 13 |
| *C/T* |  | - | 12 | 5 |
| *C/C* |  | 1 | - | - |
| g,49,315,777C>T | intergenic |  |  |  |
| *C/C* |  | - | 20 | 13 |
| *C/T* |  | - | 9 | 5 |
| *T/T* |  | 1 | - | - |
| g,49,324,268G>A | intergenic |  |  |  |
| *G/G* |  | - | 19 | 13 |
| *A/G* |  | - | 10 | 5 |
| *A/A* |  | 1 | - | - |
| g,49,346,095G>A | intergenic |  |  |  |
| *G/G* |  | - | 20 | 13 |
| *A/G* |  | - | 9 | 5 |
| *A/A* |  | 1 | - | - |
| g,49,552,834G>A | *PKHD1*, intron 50 |  |  |  |
| *G/G* |  | - | 1558 | 23 |
| *A/G* |  | 4 | 251 | - |
| *A/A* |  | 17 | 2 | - |
| g,49,565,643C>T | *PKHD1*, intron 48 |  |  |  |
| *C/C* |  | - | 30 | 19 |
| *C/T* |  | - | 4 | 4 |
| *T/T* |  | 4 | 1 | - |
| g,49,585,132A>G | *PKHD1*, intron 45 |  |  |  |
| *A/A* |  | 1 | 31 | 14 |
| *A/G* |  | - | 7 | 8 |
| *G/G* |  | 6 | 3 | 1 |
| g,49,586,767A(7_8) | *PKHD1*, intron 45 |  |  |  |
| *7/7* |  | - | 30 | 14 |
| *7/8* |  | - | 3 | 9 |
| *8/8* |  | 4 | 2 | 1 |
| g,49,589,850C>A | *PKHD1*, intron 43 |  |  |  |
| *C/C* |  | - | 28 | 14 |
| *A/C* |  | - | 3 | 4 |
| *A/A* |  | 3 | 1 | - |
| *g,49,595,529C>A* | *PKHD1*, intron 43 |  |  |  |
| *C/C* |  | - | 28 | 14 |
| *A/C* |  | - | 3 | 4 |
| *A/A* |  | 3 | 1 | - |
| g,49,596,352C>G | *PKHD1*, intron 43 |  |  |  |
| *C/C* |  | - | 31 | 14 |
| *C/G* |  | 1 | 7 | 8 |
| *G/G* |  | 6 | 3 | 1 |
| *g,49,597,760A>T* | *PKHD1*, exon 43 |  |  |  |
| *A/A* | c.6845T>A | - | 1794 | 188 |
| *A/T* | p.I2282N | 1 | 300 | 45 |
| *T/T* |  | 24 | 4 | 1 |
| *g,49,598,534G>T* | *PKHD1*, intron 41 |  |  |  |
| *G/G* |  | - | 31 | 15 |
| *G/T* |  | 1 | 7 | 7 |
| *T/T* |  | 6 | 3 | 1 |
| g,49,599,964G>A | *PKHD1*, intron 41 |  |  |  |
| *G/G* |  | - | 31 | 14 |
| *A/G* |  | 1 | 7 | 8 |
| *A/A* |  | 6 | 2 | 1 |
| *g,49,612,588_90AAGdel* | *PKHD1*, intron 37 |  |  |  |
| *AAG/AAG* |  | - | 31 | 14 |
| *AAG/del* |  | 1 | 7 | 8 |
| *del/del* |  | 6 | 3 | 1 |
| g,49,612,998G>A | *PKHD1*, intron 37 |  |  |  |
| *G/G* |  | - | 30 | 14 |
| *A/G* |  | 1 | 7 | 8 |
| *A/A* |  | 6 | 3 | 1 |
| *g,49,614,924A>G* | *PKHD1*, intron 37 |  |  |  |
| *A/A* |  | - | 27 | 14 |
| *A/G* |  | - | 3 | 4 |
| *G/G* |  | 2 | 1 | - |
| g,49,615,051G>A | *PKHD1*, intron 37 |  |  |  |
| *G/G* |  | - | 28 | 13 |
| *A/G* |  | - | 2 | 5 |
| *A/A* |  | 2 | 1 | - |
| g,49,616,787A>G | *PKHD1*, intron 37 |  |  |  |
| *A/A* |  | 1 | 28 | 14 |
| *A/G* |  | - | 2 | 4 |
| *G/G* |  | 1 | 1 | - |
| *g,49,618,202A>G* | *PKHD1*, intron 37 |  |  |  |
| *A/A* |  | - | 31 | 14 |
| *A/G* |  | 1 | 7 | 8 |
| *G/G* |  | 6 | 3 | 1 |
| g,49,621,117T>C | *PKHD1*, intron 37 |  |  |  |
| *T/T* |  | - | 28 | 14 |
| *C/T* |  | - | 2 | 4 |
| *C/C* |  | 2 | 1 | - |
| g,49,621,579G>C | *PKHD1*, intron 37 |  |  |  |
| *G/G* |  | - | 28 | 14 |
| *C/G* |  | - | 2 | 4 |
| *C/C* |  | 2 | 1 | - |
| g,49,623,196G>A | *PKHD1*, intron 37 |  |  |  |
| *G/G* |  | - | 28 | 14 |
| *A/G* |  | - | 2 | 4 |
| *A/A* |  | 2 | 1 | - |
| g,49,627,122G>C | *PKHD1*, intron 37 |  |  |  |
| *G/G* |  | - | 29 | 14 |
| *C/G* |  | - | 2 | 5 |
| *C/C* |  | 3 | 1 | 1 |
| g,49,628,960G>A | *PKHD1*, intron 37 |  |  |  |
| *G/G* |  | - | 28 | 14 |
| *A/G* |  | - | 2 | 4 |
| *A/A* |  | 2 | 1 | - |
| g,49,629,011A>G | *PKHD1*, intron 37 |  |  |  |
| *A/A* |  | - | 28 | 14 |
| *A/G* |  | - | 2 | 4 |
| *G/G* |  | 2 | 1 | - |
| g,49,630,834G>A | *PKHD1*, exon 37 |  |  |  |
| *G/G* | c.6112C>T | - | 215 | 53 |
| *A/G* | p.H2038Y | 1 | 37 | 14 |
| *A/A* |  | 7 | 3 | 1 |
| g,49,634,067Adel | *PKHD1*, intron 36 |  |  |  |
| *A/A* |  | - | 28 | 14 |
| *A/del* |  | - | 2 | 4 |
| *del/del* |  | 2 | 1 | - |
| g,49,635,005C>T | *PKHD1*, intron 36 |  |  |  |
| *C/C* |  | - | 28 | 14 |
| *C/T* |  | - | 2 | 4 |
| *T/T* |  | 2 | 1 | - |
| g,49,642,269C>G | *PKHD1*, intron 36 |  |  |  |
| *C/C* |  | - | 28 | 14 |
| *C/G* |  | - | 2 | 4 |
| *G/G* |  | 2 | 1 | - |
| g,49,644,222C>T | *PKHD1*, intron 36 |  |  |  |
| *C/C* |  | - | 31 | 14 |
| *C/T* |  | 1 | 7 | 8 |
| *T/T* |  | 6 | 3 | 1 |
| g,49,651,537A>T | *PKHD1*, intron 35 |  |  |  |
| *A/A* |  | - | 30 | 18 |
| *A/T* |  | - | 3 | 5 |
| *T/T* |  | 4 | 1 | 1 |
| g,49,651,605T>C | *PKHD1*, intron 35 |  |  |  |
| *T/T* |  | - | 29 | 14 |
| *C/T* |  | - | 3 | 9 |
| *C/C* |  | 4 | 1 | 1 |
| g,49,659,758G>A | *PKHD1*, intron 35 |  |  |  |
| *G/G* |  | - | 30 | 18 |
| *A/G* |  | - | 3 | 5 |
| *A/A* |  | 4 | 1 | 1 |
| g,49,660,275T>C | *PKHD1*, intron 35 |  |  |  |
| *T/T* |  | - | 31 | 18 |
| *C/T* |  | 1 | 4 | 5 |
| *C/C* |  | 4 | 1 | 1 |
| *g,49,667,083T>A* | *PKHD1*, intron 35 |  |  |  |
| *T/T* |  | - | 31 | 18 |
| *A/T* |  | 1 | 4 | 5 |
| *A/A* |  | 4 | 1 | 1 |
| g,49,668,903G>A | *PKHD1*, intron 35 |  |  |  |
| *G/G* |  | - | 29 | 15 |
| *A/G* |  | - | 2 | 3 |
| *A/A* |  | 2 | - | - |
| g,49,676,893A>G | *PKHD1*, intron 35 |  |  |  |
| *A/A* |  | - | 31 | 13 |
| *A/G* |  | - | 9 | 9 |
| *G/G* |  | 6 | 1 | 1 |
| g,49,677,414_5ACdel | *PKHD1*, intron 35 |  |  |  |
| *AC/AC* |  | - | 24 | 12 |
| *AC/del* |  | - | 7 | 6 |
| *del/del* |  | 2 | - | - |
| g,49,678,100C>T | *PKHD1*, intron 35 |  |  |  |
| *C/C* |  | - | 31 | 17 |
| *C/T* |  | - | 7 | 3 |
| *T/T* |  | 6 | 3 | 3 |
| *g,49,682,374A>C* | *PKHD1*, intron 35 |  |  |  |
| *A/A* |  | - | 28 | 15 |
| *A/C* |  | 1 | 5 | 4 |
| *C/C* |  | 3 | 1 | - |
| g,49,690,460C>T | *PKHD1*, intron 35 |  |  |  |
| *C/C* |  | - | 30 | 18 |
| *C/T* |  | 1 | 5 | 5 |
| *T/T* |  | 4 | 1 | 1 |
| g,49,691,268G>A | *PKHD1*, intron 35 |  |  |  |
| *G/G* |  | - | 31 | 17 |
| *A/G* |  | 1 | 9 | 5 |
| *A/A* |  | 6 | 1 | 1 |
| g,49,691,715A>T | *PKHD1*, intron 35 |  |  |  |
| *A/A* |  | - | 31 | 17 |
| *A/T* |  | 1 | 9 | 5 |
| *T/T* |  | 6 | 1 | 1 |
| g,49,692,262T>A | *PKHD1*, intron 35 |  |  |  |
| *T/T* |  | - | 30 | 16 |
| *A/T* |  | 1 | 5 | 3 |
| *A/A* |  | 4 | 1 | 1 |
| g,49,693,675T>C | *PKHD1*, intron 35 |  |  |  |
| *T/T* |  | - | 28 | 15 |
| *C/T* |  | - | 3 | 3 |
| *C/C* |  | 2 | - | - |
| g,49,697,821C>T | *PKHD1*, intron 34 |  |  |  |
| *C/C* |  | - | 30 | 18 |
| *C/T* |  | 1 | 5 | 5 |
| *T/T* |  | 4 | 1 | 1 |
| g,49,698,198T>C | *PKHD1*, intron 34 |  |  |  |
| *T/T* |  | - | 30 | 18 |
| *C/T* |  | 1 | 5 | 5 |
| *C/C* |  | 4 | 1 | 1 |
| g,50,087,495C>A | *EFHC1*, intron 2 |  |  |  |
| *C/C* |  | - | 17 | 5 |
| *A/C* |  | - | 12 | 12 |
| *A/A* |  | 1 | - | - |

^a^In addition to the horses of whole genome sequencing (Table S1) individuals of the following horse breeds were used for genotyping by Sanger sequencing: American Paint horse, American Standardbred, Anglo-Arabian, Arabian Thoroughbred, Belgian horse, Black Forest Horse, Camargue horse, Clydesdale, Camarillo White horse, Irish Sport horse, Icelandic horse, Knabstrupper, Lewitzer, Miniature horse, New Forest pony, Noriker Horse, Percheron, Quarter horse, Shetland pony, Shire horse, South German Cold-Blood, Thoroughbred, Warmblood.
